# Postgraduate Operating Room Nursing Students’ Experiences with Blended Learning Combining Digital Learning Paths and Basic Skills Training as Preparation for Internship: A Qualitative Study

**DOI:** 10.1177/23779608241278541

**Published:** 2024-09-10

**Authors:** Lena Rengård Kolstad, Vibeke Tjugum, Irene Rød, Karoline Skedsmo, Hege Vistven Stenseth, Marit Hegg Reime

**Affiliations:** 1 155319 Lovisenberg Diaconal University College, Oslo, Norway; 2 Department of Health and Social Sciences, Faculty of Health and Social Sciences, 1657 Western Norway University of Applied Sciences, Bergen, Norway

**Keywords:** digital learning paths, skill training, operating room, nursing education

## Abstract

**Introduction:**

Numerous pedagogical practices ought to be contemplated for the acquisition of practical aptitudes imperative to postgraduate operating room nursing education. The employment of digital technologies has emerged as a strategic focus in higher education and learning paths exhibit potential as a digital approach in nursing education.

**Objective:**

This study aimed to investigate the experiences of postgraduate OR nursing students who underwent a blended learning approach, which combines digital learning paths with skills training, and to explore how this approach prepares students to attain specific learning outcomes during their internship period.

**Methods:**

This qualitative study employed a descriptive, exploratory design and utilized focus group interviews facilitated by an interview guide to gather qualitative data. A purposive sampling strategy was employed, and the collected data were analyzed using a systematic text condensation approach.

**Results:**

The analysis of the data revealed two main categories and five subgroups. The first category, “Blended learning serves as adequate preparation for internship,” includes subgroups that highlight the advantages of diverse learning activities that aid in the development of a strong foundation in practical skills. The positive influence of peer collaboration fosters improved learning through social interaction, while the organization of the curriculum has a significant impact on students’ learning experiences. The second category, “The importance of skills training and behaving in an operating theater context,” consists of subgroups that emphasize the necessity of progressing from basic technical skills training to simulation pedagogy to ensure appropriate behavior in the operating room. Small group sizes, close monitoring, and assessment by educators contribute to effective learning.

**Conclusion:**

The integration of digital learning paths with skills training fosters a problem-solving approach and encourages active and collaborative learning. Skills training in small groups, timely feedback, and coordination among subject managers to handle the students’ workload can create an optimal learning environment.

## Introduction/Background

In the field of operating room (OR) nursing, the acquisition of skills is a gradual process that occurs during both educational and clinical internship phases. This progression entails engaging in tailored learning activities at an appropriate taxonomical level. Postgraduate OR nursing students must first master fundamental skills, such as surgical hand disinfection, sterile dressing, patient positioning, and instrumentation, before advancing to more complex practical skills (Cuming, 2022).

To enhance the quality of OR nursing education, the application of constructive alignment as an educational principle can be beneficial (Biggs et al., 2022). The term “constructive” pertains to the learners’ active role in constructing meaning through relevant learning activities. On the other hand, the term “alignment” refers to the actions undertaken by educators (Biggs et al., 2022). Constructive alignment is an outcome-based educational approach that entails aligning crucial elements of the educational program, such as teaching and learning activities and assessment methods, with the intended learning outcomes of students (Biggs et al., 2022).

In Norway, the education of OR nurses comprise two options: postgraduate education with a duration of 18 months (90 credits, full-time studies) and a master's degree with a duration of 24 months (120 credits, full-time studies). The key distinction between these programs is the inclusion of a master's thesis worth 30 credits. As of January 2023, all educational institutions offering OR nursing education are required to adhere to the new National Curriculum Regulations for Norwegian Health and Welfare Education (Forskrift om nasjonal retningslinje for operasjonssykepleierutdanning, 2023). In line with this curriculum, a university college situated in south-eastern Norway use a blended learning strategy for their OR nursing program. This approach combines a digital learning path with skills training in the foundational OR nursing skills course during the first semester of the program.

Through various learning activities, peer learning was an educational strategy where students collaborated in small groups to meet the course's learning outcomes (Table 1). Peer learning includes collaboration, reflection, and communication between students either at the same level or at different academic levels (Josse-Eklund et al., 2023). The accumulated knowledge suggests that students in peer learning gain greater confidence and independence in learning and acquire a higher level of personal and professional skills (Josse-Eklund et al., 2023). Our project activities included reading and discussing the syllabus and recommended literature, watching procedural videos, solving assignments and quizzes, and training procedural skills together. Table 1 provides an overview of the learning activities and learning outcomes covered in this course.

**Table 1.** Learning Activities and Outcomes in the Digital Learning Path.

Learning activities	Learning outcomes - Knowledge	Learning outcomes - Practical skills	Learning outcomes - General competency
1. Reading the syllabus and the recommended literature 2. Watching videos about the procedures 3. Discussing assignments in groups 4. Completing individual assignments and quizzes	Have in-depth knowledge on: - Aseptic methods used during surgical procedures - Checking and counting instruments and operating materials - Disinfecting operating fields - Moving and positioning patients	Manage: - Surgical handwashing and disinfection - Sterile dressing and use of a mayo table - Basic instrumentation, surgical counting and drawing on a mayo table - Disinfecting and covering a square operating field - Moving patients from the bed to the operating table - Place patients in the supine and lithotomy positions	Manage: - Implementing infection prevention and applying hygienic principles - Quality assurance in terms of the preparation and use of surgical instruments and operating materials - Assessing the patient’s health and preventing complications in surgical treatment and positioning

Several studies have highlighted the limited opportunities for OR nursing students to acquire practical skills. This scarcity underscores the importance of providing extensive skills training due to the unfamiliarity of the clinical environment and the unique roles and responsibilities of OR nurses (Mafinejad et al., 2022; Prince, 2004; Sarikaya et al., 2006). As OR nursing students prepare for their first internship period, it becomes crucial for them to acquire basic skills in the most common procedures performed in the operating theater. This preparation is necessary for achieving a state of readiness, as outlined in Table 1. The learning outcomes specified in this table are closely aligned with what OR nursing students should aim to master during their initial internship period.

## Review of Literature

The use of digital technologies in higher education has become a global priority, with a focus on innovation and advancement (Sormunen et al., 2022). In line with this, the Norwegian government, in its report ‘Quality Culture in Higher Education,’ emphasizes the importance of providing stimulating and diverse learning and assessment methods that utilize digital opportunities for all students (Ministry of Education and Research, 2017).

Digital learning interventions in higher education have shown positive outcomes. They enhance students’ professional knowledge, skills, and attitudes, while also improving their academic performance, collaborative abilities, and study skills (Button et al., 2014; Hernon et al., 2023; Männistö et al., 2020; Sormunen et al., 2022). Additionally, digital learning has been linked to increased professional confidence and self-efficacy in clinical skills, problem-solving, decision-making, teaching and counseling, and professional communication skills (Sormunen et al., 2022). Overall, the integration of digital learning methods in higher education has proven to be beneficial for students, empowering them with a comprehensive set of skills and improving their overall educational experience. Numerous studies have demonstrated that blended learning in health education yields more favorable outcomes compared to traditional learning methods (Lee & Park, 2018; Vallée et al., 2020). Blended learning involves a combination of traditional face-to-face learning and either asynchronous or synchronous e-learning (Vallée et al., 2020). In a digital learning path, students progress through learning activities in alignment with the descriptions of the intended learning outcomes (Biggs et al., 2022). Digital learning paths are closely interconnected with the concept of active learning. Active learning requires students to actively process, reflect upon, and apply the content based on their own preexisting knowledge and the professional context (Prince, 2004).

Digital learning paths make teaching more dynamic, engaging, and beneficial for students (Welch Bacon & Gaither, 2020). By activating multiple senses, digital learning paths acknowledge that students have different learning strategies and styles, and that employing a variety of teaching methods enhances motivation to learn (Repstad & Tallaksen, 2006). These digital learning paths provide students with the opportunity to construct new knowledge upon their existing knowledge. When new knowledge is integrated with preexisting knowledge, learning and retention are enhanced (Piaget & Inhelder, 1969; Vygotsky, 1978).

Digital learning paths offer the potential to enhance learning outcomes across various disciplines and mitigate barriers related to resources, geographical limitations, and time constraints. This, in turn, contributes to a more flexible education system (Fossland, 2015). The emergence of diverse learning platforms, such as Learning Management Systems and Virtual Learning Environments, further facilitates flexibility for students and provides a wider array of learning and assessment methodologies for OR nursing students (Fossland, 2015; Meum et al., 2021). The terms e-learning, online learning, virtual learning, web-based learning, and distance learning all encompass various forms of digital learning. Studies comparing traditional learning methods with digital learning methods have consistently shown that digital learning methods are advantageous in terms of skills performance and higher content retention (Abarghouie et al., 2020) and the development of self-leadership and problem-solving skills (Lee & Park, 2018). Furthermore, e-learning resources have been found to enhance preparedness among medical students, undergraduate nursing students, postgraduate OR nursing students and anesthesiology nursing students before their practice in the OR, with videos being identified as the most beneficial resource (Fagerdahl et al., 2021). In addition, e-learning has been shown to increase knowledge regarding patient moving, transferring, and positioning among OR nurses (Khorammakan et al., 2024). A blended learning course focusing on minimally invasive surgery for nurses has also demonstrated potential in bridging the training gap for nurses working in different countries (Ortega-Morán et al., 2021).

Despite these positive findings, there is a scarcity of information regarding the use of blended learning which combines digital learning paths with skills training in the field of postgraduate OR nursing education. This research gap justifies the need for further investigation in this area. Thus, this study aimed to investigate the experiences of postgraduate OR nursing students who underwent a blended learning approach combining digital learning paths with skills training. The main objective was to explore how this approach prepares students to successfully attain specific learning outcomes during their initial internship period.

## Methods

### Design

**Figure 1.**
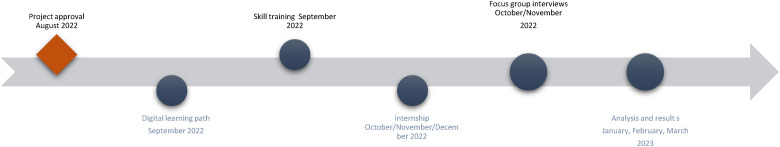
Study timeline template.

This research employed a qualitative descriptive exploratory design, utilizing focus group interviews as the primary method of data collection (Hunter et al., 2019). The use of focus groups is well-suited for investigating human characteristics such as experiences, thoughts, motives, and attitudes (Malterud, 2012). Qualitative research aims to understand rather than explain and to describe rather than predict (Malterud, 2017). Given the limited number of studies exploring student experiences with blended learning approaches in postgraduate OR nursing education, this design was deemed appropriate (Hunter et al., 2019). Furthermore, this study adhered to the Consolidated Criteria for Reporting Qualitative Research guidelines, ensuring transparent reporting of the research process (Tong et al., 2007). Figure 1 demonstrates the timeline template for the study, outlining the various stages and activities conducted.

### Research Question

What are the experiences of postgraduate OR nursing students with a blended learning approach that integrates a digital learning path with skills training, and how does this approach prepare them for their internal internship?

### Participants and Setting

The study took place at a university college in southeastern Norway, which offers postgraduate and master's degree programs in OR nursing education. A purposive sampling strategy was utilized to recruit postgraduate students enrolled in this program (Polit & Beck, 2017). All students (*n* = 41) enrolled in the postgraduate OR nursing educational program were given detailed information about the study through both written and oral means. The first author personally delivered the information in the classroom and also sent out information via email. Interested students who wished to participate were instructed to email the first author. A total of 17 female and 1 male student agreed to take part in the focus group interviews.

### Data Collection

Two focus groups with nine participants in each group were conducted four weeks after the skills training session. At this point, the students had started their eight-week internship period. The focus groups utilized a semistructured interview guide (Table 2), which consisted of a few open-ended questions to facilitate discussions and exchange of opinions among the students (Kvale & Brinkmann, 2009; Malterud, 2012). If needed, follow-up questions were posed to ensure comprehensive coverage of the topic. The interview guide was developed in collaboration with several of the authors but was not pilot-tested prior to the study.

**Table 2.** Interview Guide.

Main questions	Follow-up questions
**Can you describe how you experienced the digital learning path and skills training in preparation for the first internship period?**	Can you describe this in more detail? Can you elaborate on this? Can you give an example? Can you link this experience to a specific part of the learning path? Does anyone else want to add anything?
**Can you tell us about how you collaborated with other students during the digital learning path and skills training?**	Can you describe this in more detail? Can you elaborate on this? Can you give an example? Can you link this experience to a specific part of the learning path and skills training? Does anyone else want to add anything?
**Can you give us feedback on points for improvement in the curriculum?**	Can you elaborate on this? Can you give an example?

The focus group interviews were led by one moderator and one comoderator who were both trained in conducting this type of interview. The interviewers were educators who were not directly involved in the operating nursing program, allowing the students to freely express their opinions. The interviews took place at the university college and had an approximate duration of one hour each. All the interviews were digitally recorded and subsequently transcribed verbatim by the first author, resulting in a transcription material of 41 pages in Microsoft Word format. Participant validation of the transcripts was not performed.

### Institutional Review Board Approval

The study obtained ethical approval from the Norwegian Agency for Shared Services in Education and Research (no. 878323) and from the head of the department at the university college. Written consent forms were distributed to the students, and they were required to sign these forms before participating in the interviews. It was assured that participation or nonparticipation in the study would not have any impact on the students’ future studies, and they had the right to withdraw from the study at any time without facing any negative consequences. The study followed ethical guidelines and standards outlined in the Declaration of Helsinki, including obtaining written informed consent, ensuring the right to withdraw, and guaranteeing anonymity for the participants (Shrestha & Dunn, 2019; The World Medical Association, 2022).

### Data Analysis

The analysis of the data was conducted by three authors (LK, VT, and MHR) using the systematic text condensation (STC) method, as described by Malterud (2012), which draws inspiration from Amedeo Giorgi's psychological–phenomenological method (Malterud, 2017). Systematic text condensation, similar to phenomenology, aims to understand the subjective experiences of the participants as the basis for generating knowledge.

The analytic process consisted of four steps. In the first step, each researcher independently read through the entire dataset and identified initial themes by taking a broad, overall view of the material. In the second step, meaning units were identified, and the researchers organized these units into codes to create an analytical framework that allowed for potential nuances.

Coding involved systematically decontextualizing the text, extracting portions of the text from its original context, and grouping them with related elements in the light of theoretical perspectives (Malterud, 2012, 2017). In this step, the initial themes were refined through discussions among the authors, resulting in two main code groups. In the third phase, each code was further divided into subgroups, and illustrative quotations from the interviews were selected for each subgroup. To condense the data, artificial quotes (artifacts) were created to represent the content of the individual meanings within each subgroup.

In the fourth phase, the extracted elements were recontextualized by integrating them back into their original context and summarized in the form of interpretive syntheses. This process aimed to provide an overall understanding of the data and generate descriptive and conceptual insights that captured the essence of the participants’ experiences. An example of this process is outlined in Table 3.

**Table 3.** Example of the Analytical Process.

Code group	Meaning units (selected)	Subgroups	Condensate	Synthesis
**The digital learning path in preparation for skills training**	*Yes, the digital learning path was especially nice because everything was new, and we did it in groups. Then, we got to talk a bit about it and hear what other people thought. Yes, you are able to learn from other people's thoughts.* *We had good conversations in our group. We had discussions and were very much together when answering assignments.*	Collaboration with peers promotes enhanced learning through social interaction	‘I learn a lot from what other people think’ (2-FG2). ‘Working together made me get to know the others better. I think it's good that I have someone else who supports me in a way” (2-FG1). “I became more active and involved when we had to solve the tasks together” (6-FG2). Golden quote: ‘By solving tasks and quizzes in the learning path together, I took more responsibility for each other's learning. Working together in groups provided a significant amount of learning, many good discussions and close cooperation.’	Solving tasks together in groups encourages students to take more responsibility for each other's learning. Group work strengthens social cohesion and communities of fellow students.

Throughout the analysis, the researchers engaged in ongoing discussions to ensure consensus and enhance the reliability and validity of the findings. By including excerpts and quotes from the interviews, the authors aimed to illustrate and support the main themes identified during the analysis.

### Rigor

Ensuring trustworthiness is crucial in qualitative research to maintain reliability and validity (Jayasekara, 2012). Although the terms *reliability* and *validity* are essential criteria for quality in quantitative paradigms, in qualitative paradigms the terms *credibility*, *neutrality* or *confirmability*, *consistency* or *dependability*, and *applicability* or *transferability* are to be the essential criteria for quality (Jayasekara, 2012).

In this study, several actions were taken to increase trustworthiness. Credibility was enhanced by recruiting participants who were capable of answering the research question effectively. Additionally, during the analysis process, coding of the data was discussed multiple times among three of the authors, and input from other researchers was sought in the final phase to ensure credibility of the findings (Polit & Beck, 2017). As the first author was an OR nurse educator, efforts were made to ensure that interpretations were derived from the data itself, further strengthening confirmability (Nowell et al., 2017). Dependability was ensured by thoroughly documenting the research process and using the same semistructured interview guide in both focus groups. This consistency reduced the potential for variation in data collection and analysis (Jayasekara, 2012). To enhance transferability, a comprehensive description of the participants, the setting, the digital learning path, and the skills training program, as well as the research process, was provided. The use of quotations from the participants and the discussion of findings in relation to other international studies also contributed to the trustworthiness of the study (Jayasekara, 2012).

Reflexivity, which involves critically reflecting on one's role as a researcher and its influence on the research process, was addressed by the first author through written reflexivity notes. These notes described the choices and considerations made during the analysis process, providing transparency and an opportunity to identify potential preconceptions that could have impacted the analysis (Malterud, 2017). Collectively, these actions taken to enhance trustworthiness contribute to the overall rigor and quality of the study.

## Results

### Participant Characteristics

The participants mean age was 34.7 years. To apply for the postgraduate OR nursing education, registered nurses must have a minimum of 2 years’ work experience. In this sample, registered nurses had a mean of 8.6 years of work experience before attending the postgraduate OR nurse education (Table 4).

**Table 4.** Description of the Participants.

Variables	Focus group 1 (*n* = 9)	Focus group 2 (*n* = 9)	Total sample (*n* = 18)
Gender			
Male	0	1	1
Female	9	8	17
Age			
Median (range)	33 (28–52)	34 (27–40)	33.5 (27–52)
Mean (SD)	35.9 (8.62)	33.6 (4.47)	34.7 (6.97)
Experience as registered nurse (years)			
Median (range)	7 (4–20)	7 (5–15)	7 (4–20)
Mean (SD)	8.9 (5.48)	8.3 (3.65)	8.6 (4.69)

### Research Question Results

From the analysis of the data, two main categories and five subgroups were identified (Table 5). These categories and subgroups provide a comprehensive understanding of the experiences of postgraduate OR nursing students. The presence of many similarities and few differences between the two focus groups indicates that a comprehensive understanding of the experiences of postgraduate OR nursing students has been achieved, and further data collection may not yield significantly different insights.

**Table 5.** Main Categories and Subgroups.

Main categories	Subgroups
Blended learning serve as adequate preparation for internship	Various learning activities help develop the understanding of the foundation for practical skill performance Collaboration with peers promote enhanced learning through social interaction The organization of the curriculum affects students learning
2. The importance of skills training and behaving in an operating theater context	Desire for taxonomical progression from basic technical skills training to simulation pedagogy to train correct behavior in the operating room Small group sizes and close follow-up and assessment from educators facilitate learning

### Main Category 1: Blended Learning Serve as Adequate Preparation for Internship

#### Subgroup 1: Various Learning Activities Help Develop the Foundation for Practical Skill Performance

The various learning activities in the digital learning path were perceived to give the OR nursing students a theoretical understanding of skills important for the field and were helpful as preparation before training basic practical skills. The focus on basic skills was perceived as helpful because the students had no previous experience in OR nursing or knowledge of required competences. They described the content of the learning path as relevant and that attention to basic skills was important for their professional development to master basic skills as OR nurses.

Everything was relevant and very useful to understanding how and why we should perform the skills. (Student 1-FG1)

I'm glad I had it before the skills training because then I could both see how it should be done and read in advance what the theoretical background was. (Student 2-FG1)

The students emphasized that the theoretical topics in the digital learning path provided them with a deeper understanding when it comes to training practical skills. Thus, they could begin the training session at a higher taxonomic level. It was highlighted that the various learning activities in the learning path prepared them for the upcoming internship period.

The digital learning path prepared me for both skills training and practice. I was more prepared to master the basic skills I needed before the internship period. I read about the basic skills and saw them on videos; therefore, it was easier for me to accomplish these tasks when I had to do them myself. I now understood more clearly how and why those in the video perform the skills this way. (Student 5-FG1)

During the skills training, the students experienced being able to discuss their theoretical understanding with the educators and fellow students and thus gain a deeper understanding of the topic.

I think that the learning path was very preparatory for the skills training. Yes, because there were some tasks we were unsure about. However, we got more answers when we had the skills training. We were able to ask the educators questions along the way and discuss various issues. (Student 6-FG1)

#### Subgroup 2: Collaboration with Peers Promote Enhanced Learning Through Social Interaction

The digital learning path was arranged for students to collaborate in group activities with fellow students, with the opportunity to discuss and solve tasks together. The groups themselves decided when and where they wanted to carry out the digital learning path. Working together in groups was deemed a positive experience because it allowed students the opportunity to organize collaborative work based on the needs of the whole group according to requirements to be met. The students emphasized that collaboration and discussions about different topics and tasks promoted their learning outcomes. Working in groups helped to strengthen social cohesion and provide a community for the students.

It allowed me to get to know the others better. I think it's good that I have someone else who supports me in a way. (Student 2-FG1)

Then we could talk about the tasks and hear what others think. I learn a lot from what other people think. (Student 2-FG2)

The students also found that collaboration in groups made them feel more responsible for participating and more responsible for each other's learning.

It was a very nice way to get to know each other and connect. Some know a little about one thing, and others know something else. And then we manage to figure things out together. We sort of take responsibility for each other. (Student 8-FG2)

Students, to which Norwegian was their second language, expressed that collaboration with peers had even a greater positive consequence for them, as it led to a better understanding of the assignments. This implies that individual studying can involve more difficulties and possibilities of misunderstanding due to language problems.

It was nice to work together because when you sit alone and read, it's so easy to misunderstand, at least for me, who struggles with the language. (Student 2-FG1)

The students described how the organization of the digital learning path contributed to active learning. Student groups solved the tasks in the learning path slightly differently. Most of the groups solved all the tasks together, but some groups divided the tasks between them and only solved the quizzes together. Respondents from groups who organized the work as the latter expressed that they probably would have learned more if they had worked together on solving the tasks.

My group worked on the tasks together on the first day, but later we divided the tasks between us because of the time pressure and other exams. But it wasn’t as it was intended with discussions and such. I learn more when we work together. (Student 2-FG1)

Students who worked closely with their peers stated that they became more active and involved themselves.

There were many discussions, even between groups at times, so everyone became very active. (Student 7-FG1)

I think those quizzes were smart because you had to know a bit about different subjects. We went through the quizzes together, but the fact that we had to physically answer them individually, I think, is an advantage, because then it wouldn’t be just one person doing it in the group. (Student 8-FG2)

#### Subgroup 3: The Organization of the Curriculum Affects Students Learning

Some students perceived the organization and structure of the course suboptimal as preparation for the upcoming internship period. They expressed that in their experience the basic skill training came too early in the semester and the skills were hard to recall when they started their internship period several weeks later. To keep the skills and procedures fresh in their minds, the students suggested that basic skills training should be organized closer to the internship period.

The learning path and skills training should have been conducted closer to practice; I had kind of forgotten everything when I started practice. (Student 9-FG2)

The students experienced that the organization of the semester, with several subjects that were not planned in relation to workload and exams, influenced their learning. They had to prioritize studying for the exam and had not actively used the blended learning opportunities to be prepared for the upcoming internship period.

The exam took the focus away from skills training. It kind of came at the same time as we were preparing for exams in other subjects. (Student 7-FG2)

### Main Category 2: The Importance of Skills Training and Behaving in an Operating Theater Context

#### Subgroup 1: Desire for Taxonomical Progression from Basic Technical Skills Training to Simulation Pedagogy to Develop Correct Behavior in the OR

Training on basic skills was described by the students as very useful. There was a consensus from the respondents that they wanted more of such training. Although it was considered as basic skills in OR nursing, the respondents expressed that all the technical skills were experienced as complicated. Skill training with the possibility of repetition gave them a sense of mastery at a higher level compared to having no practical training before the internship period.

The skill training made me more confident about basic procedures. Even though I failed a little, I experienced mastery. (Student 4-FG2)

Furthermore, it emerged that the students thought there was too much focus on the achievement of individual technical skills compared to collaborative and nontechnical skills. They expressed a need for more training on how to behave in the operating theater, especially areas such as handing over sterile equipment, and how to behave and communicate in the operating theater. They expressed that this lack of experience posed a challenge in their introduction to the OR environment in the first internship period. The students asked for simulation pedagogy to obtain an impression of how they should behave in an OR context. They wanted several procedures and skills to be put together to prepare them for how an OR really works.

Maybe we could simulate after skills training so that we can obtain an understanding of what it is like in the operating room and not just perform one procedure at a time. (Student 4-FG2)

More attention to the use of surgical instruments and how these should be handed over to the surgeon in the OR was requested by the respondents. The basic skills training lacked information about which instruments were used for various procedures and operations. This was knowledge that the students expressed would be helpful for their first internship. This was also the skill that most students expressed that they feared failing in during the upcoming internship period.

#### Subgroup 2: Small Group Sizes and Close Follow-up and Assessment from Educators Facilitates Learning

Students expressed that the organization of the basic skills training influenced learning. They expressed the importance of thorough educational assessments to facilitate learning. Skills training in large groups were experienced to give less learning than expected. An example given by the students was related to training on techniques for sterile handwashing and disinfection. Only one sink was available for every 10 students, resulting in idle time while waiting for access to training. Thus, the students expressed that large groups were associated with fewer opportunities to practice skills and repetitive training as well as less access to the educators. With the large groups, the feedback provided from educators was impaired by the number of students and limited time.

I learned a lot when we were in small groups and could practice again and again. (Student 1-FG2)

The students highlighted that feedback and corrections from educators in skills training were important for effective learning. Without feedback, they became unsure whether they had performed the procedures correctly, and the exercise was perceived as less meaningful. One student said:

No one could tell if I made a mistake; then, there was no point in practicing. (Student 5-FG2)

Training practical skills in small groups was however experienced as conducive to learning. Advantages reported by the students were that small groups provided less noise, less waiting, and better opportunities to repetitive training. The students expressed that they preferred small groups and the presence of several educators, with the possibility of feedback. Skills training organized in this way can thus promote and improve learning compared to large groups.

## Discussion

This study aimed to investigate the experiences of postgraduate OR nursing students who underwent a blended learning approach, which combines digital learning paths with skills training. The main objective was to explore how this approach prepares students to successfully attain specific learning outcomes during their initial internship period. The students emphasized that the use of the digital learning path with different educational methods promoted active and collaborative learning and helped students to develop the foundation for practical skills development and served as good preparation for skills training. Learning together, using the peer-learning method in a digital learning environment has been shown to have encouraging effects for enhancing students’ knowledge, competence, satisfaction, and problem-solving skills (Josse-Eklund et al., 2023; Männistö et al., 2020). Our findings support the idea that digital technologies enable increased availability of knowledge and teaching resources and thus facilitate a learner-centric approach to teaching, adding value to learning processes (Meum et al., 2021). In addition, the students appreciated being able to conduct learning activities whenever it suited them. This is in accordance with more flexible solutions found in higher education, supported by several studies dealing with digital learning methods for students (Fossland, 2015; Meum et al., 2021). The various activities in the digital learning path were found to be useful both in preparation for students’ skills training and as preparation before the upcoming internship period. Students emphasized that the videos and the opportunity to familiarize themselves with the theory provided them with a deeper understanding of the foundation for skills development, enabling them to start at a higher taxonomic level before skills training and the internship period. This is consistent with the findings from Fagerdahl et al. (2021) revealing that videos made the students feel more prepared and relaxed when attending the OR. The variety of tasks in the digital learning path, such as reading research articles, solving tasks, taking quizzes, and watching videos together in groups, contributed to a deeper understanding of why basic skills were carried out in the way they were. This is also in line with Smeby and Heggen (2014), who emphasized the importance of coherence in the educational program to integrate theory and practice. However, designing learning paths in a way that leads the students through different learning activities to achieve learning outcomes may be challenging for educators, as it requires time and skills to incorporate new technology that emerges continuously (Button et al., 2014; Hernon et al., 2023).

Both the digital learning path and the skills training program facilitated active and collaborative learning. Discussing and solving tasks together in the digital learning environment were experienced as positive and promoted learning. The students found that the discussions they had together yielded the most learning because they learned a lot from listening to fellow students’ points of view. This can be seen in relation to the transformative learning theory, which emphasizes that the goal of adult education is to help individuals become more autonomous thinkers by learning to negotiate their own values, meanings, and purposes rather than to uncritically act on those of others (Mezirow, 1997). Critical reflection and participation in discourses thus become significant elements in higher education pedagogy (Mezirow, 1997).

In the present study, OR nursing students experienced that the learning path helped them to strengthen social cohesion. Through group work, they were able to acquaint themselves with their fellow students and receive support from each other, which resulted in a sense of community. Similarly, a systematic review of randomized control trials also highlighted that digital collaborative learning environments contributed to interaction skills and problem-solving skills, in addition to satisfaction and motivation for learning (Sormunen et al., 2022). Besides, another systematic review and meta-analysis found that peer-assisted learning benefited academic performance and clinical skills performance (Brierley et al., 2022). Social support is likelier to occur when social interaction is a dominant type of learning activity (Berings et al., 2008; Männistö et al., 2020). Groups that solved all the tasks and quizzes in the digital learning path together took more responsibility for each other's learning. This was particularly important for students whose second language was Norwegian, as group work helped them increase their understanding of the subjects*.* Previous studies have shown that second-language nursing students must learn three languages in parallel: Norwegian, academic concepts, and nursing terminology (Garone et al., 2020). In addition, second-language nursing students struggles with both oral and written assignments, the interpretation of assigned texts and the systematization of content in their own texts (Amaro et al., 2006; Black & MacKenzie, 2008; Crawford & Candlin, 2013)*.* These findings are important for nurse educators to consider when designing learning activities.

Groups that divided the tasks between them due to time constraints and only solved the quizzes together reported lower learning acquisition than those who fully collaborated through the digital learning path. However, earlier studies have shown that experiences with group work, such as free-riding, challenging group processes, group sizes, and types of tasks impact satisfaction with group work (Chang & Kang, 2016). According to social cognitive learning theory, learning is achieved when students are active and interact with those around them (Locke, 1987). Educators in higher education have a responsibility to facilitate adult students’ functioning as more autonomous and socially responsible thinkers. This requires communicative learning; therefore, group work may contribute to such learning (Mezirow, 1997).

Some of the students stated that the basic skills training program came too early in the semester, leading to low skill retention in relation to their upcoming internship period. In addition, the semester schedule contained several parallel subjects to attend for the students, with a perceived high total workload. The students then prioritized studying for their exams in other subjects and did not work on the digital learning path as much as planned. These findings show the major impact exams pose on students’ priorities. To prevent students themselves having to set such priorities, this finding calls for educators to cooperate on student's workload and exams when planning the academic year for various subjects (Kyndt et al., 2016). This is in line with constructive alignment theory, which specifies that educators should consider the overall organization of subjects to facilitate optimal learning conditions (Biggs et al., 2022).

Training on practical basic skills provided students with confidence, even if they occasionally failed. In accordance with deliberate practice, skills training with the opportunity for repetition and feedback allows students to experience mastery at a higher level than if they had no prior experience (Ericsson et al., 1993; Ericsson & Harwell, 2019). Deliberate practice suggests that organizing skills training around learning goals, rehearsal and feedback loops are essential to develop highly skilled practitioners (Donoghue et al., 2021; Welch & Carter, 2018). In the present study, the students wanted more training on skills because such training gave them self-confidence. In addition, the students expressed that they wanted more training on how to behave in an OR environment. Handing over instruments to the surgeon was described as one of the skills most students were afraid of doing wrong, congruent with findings from Fagerdahl et al. (2021) who argued that stress and anxiety among students may inhibit learning. According to Benner (2010) complex skills must be learned in authentic clinical learning environments. Our students called for increased use of simulation-based learning to present them with learning opportunities in an environment that replicates an operating theater. The opportunity to apply technical and nontechnical skills in a realistic environment can prepare students for their professional roles as OR nurses. Research reveals that regular interprofessional simulation-based training to expand the repertoire of situations students are likely to encounter in clinical practice is in demand (Kaldheim et al., 2021). However, students want to start learning basic technical skills before moving into more advanced interprofessional training, seeking to build new knowledge on top of their existing knowledge (Kaldheim et al., 2021). For OR nursing education, it is important to be able to create coherence and help students recontextualize the knowledge they have developed at school to another learning arena, such as the operating theater (Smeby & Heggen, 2014).

Varying group sizes affected the learning outcomes of the skills training. In large groups with many students, there was a significant amount of waiting, fewer opportunities to practice, and weakened feedback from educators. Consequently, smaller groups were more conducive to learning and provided less noise, less waiting, and better opportunities to practice several times. Although this skills training program was not subject to examination or testing, some of the students expressed that there was no point in practicing if no one gave them feedback. This shows that feedback is a critical component of effective tutoring, as feedback facilitates student learning and performance improvement (Weallans et al., 2022). According to the study by Männistö et al. (2020), educators play a crucial role in providing feedback to strengthen students’ self-confidence and mastery of skills. It is therefore important for education programs to have enough teachers present to be able to provide timely feedback.

### Strengths and Limitations

No previous research exploring how postgraduate OR nursing students experience the use of digital learning paths combined with skills training were found, which strengthens the relevance of this study. However, some limitations must be addressed.

Various suggestions exist for optimal participant numbers in focus groups, but most authors suggest that an adequate group size should involve 4–12 participants, with the optimal size being between 5 and 10 (Krueger & Casey, 2015; Morgan, 1996; Sim, 1998). The group must be large enough to provide comprehensive data, but small enough for everyone in the group to be heard. However, the focus group size may have contributed to follow-up questions not being asked to further elaborate a deeper understanding of their statements. The experience from the present study is that the data were rich and that all the participants were active. Nevertheless, there is a possibility that some students did not share everything they wanted because the groups were relatively large, with nine participants in each group. Jayasekara (2012) claims that the real strength of focus groups is the insights given into the sources of complex behaviors and motivations, in addition to exchange of opinions by discussing both common experiences and unique experiences (Malterud, 2012). This corresponds with the purpose of our study exploring OR nursing students’ experiences with the use of digital learning paths combined with skills training.

Leentjens and Levenson (2013) raised ethical issues about the recruitment and inclusion of students in university research projects because students may be required or coerced to participate, violating their privacy. In our study, the first author who invited the students to participate in the study was an OR nurse educator, which may have led to students feeling obliged to participate. However, the moderator and the comoderator were educators from other disciplines; thus, the students were encouraged to speak more freely. The moderators had experience with digital learning paths but were not familiar with the OR context. This may have led to natural contextual follow-up questions being omitted.

### Implications for Practice

The findings suggest that a blended learning approach may enhance students’ ability to achieve defined learning outcomes. However, several important variables influencing the students’ learning process must be addressed. The use of small groups with close follow-up by the educators’ during skills training was highlighted by the students as one important element that could increase students’ learning outcomes. Besides, students called for more simulation-based training on how to behave in the operating theater in a professional manner. In addition, virtual reality may also offer an alternative clinical experience to physical simulation to increase the students’ level of confidence (Sen et al., 2022; Siah et al., 2022). Furthermore, training practical skills should appear closer to the internship period to facilitate the transfer of skills. Better cooperation between educators on student's workload and exams when planning the academic year for various subjects may also facilitate better learning outcome for the students. Educators should take these factors into consideration when organizing education for the OR nursing students.

## Conclusion

Using digital learning paths and basic skills training as pedagogical tools to achieve defined learning outcomes can lead to more effective and deeper learning for postgraduate OR nursing students. However, there is a high demand for training in operating theater behavior before OR nursing students’ internship periods. Supplementing skill training with simulation training in a realistic clinical environment may better prepare OR nursing students for internship periods. Feedback and corrections from educators, in addition to sufficient time and smaller groups, are essential elements in high-quality skills training. Educators must intensify collaboration within various subjects to plan the students’ academic year with the aim of optimizing the student's learning outcomes. New technologies, such as virtual reality, may also contribute to student's clinical experiences in a future curriculum.

## Supplemental Material

sj-docx-1-son-10.1177_23779608241278541 - Supplemental material for Postgraduate Operating Room Nursing Students’ Experiences with Blended Learning Combining Digital Learning Paths and Basic Skills Training as Preparation for Internship: A Qualitative Study Supplemental material, sj-docx-1-son-10.1177_23779608241278541 for Postgraduate Operating Room Nursing Students’ Experiences with Blended Learning Combining Digital Learning Paths and Basic Skills Training as Preparation for Internship: A Qualitative Study by Lena Rengård Kolstad, Vibeke Tjugum, Irene Rød, Karoline Skedsmo, Hege Vistven Stenseth and Marit Hegg Reime in SAGE Open Nursing
